# A Novel Approach to the Design and Sample Size Planning of Animal Experiments Based on Effect Estimation

**DOI:** 10.1002/bimj.70116

**Published:** 2026-03-15

**Authors:** Dario Zocholl, Henrike Solveen, Matthias Schmid

**Affiliations:** ^1^ Institute of Medical Biometry Informatics and Epidemiology Medical Faculty University of Bonn Bonn Germany

**Keywords:** animal experiments, design of experiment, effect estimation, multiple testing, sample size planning

## Abstract

Animal experiments are often purely exploratory, with little to no data available to support the planning phase. Nonetheless, ethical guidelines demand scientifically sound planning, particularly regarding sample size determination based on biometric criteria such as power analysis or precision of effect estimation. The experimental designs are typically complex, involving numerous experimental groups and adaptive steps, which complicates statistical planning. To date, existing statistical approaches for animal experiments have largely ignored this complexity. Despite widespread recognition that effect sizes in animal studies are often biased, poorly replicable, and rarely translate well to clinical trials, little emphasis has been put on this remarkable gap between experimental research and statistical planning. We demonstrate that common design practices in animal experiments introduce substantial error in effect size estimation, even if properly adjusted for inflated type I error rates and false discovery rates. To address this, we propose a simulation‐based approach to quantify the estimation error and to classify its magnitude compared to a reference design. We advocate for a two‐stage experimental approach—comprising a screening and a confirmation phase—using robust mixture priors for effect size estimation. Our simulation study compares the operating characteristics of various designs and illustrates how optimal designs can be selected. Additionally, we present supporting software tools aimed at facilitating communication with nonstatistical collaborators.

## 1 Introduction

In preclinical biomedical research, animal experiments play a critical role in exploring physiological mechanisms, disease models, and therapeutic interventions. Within the European Union, more than 9 million animals (of which 55% were mice or rats) were used for research and testing in 2022 (European Commission Summary Report on the statistics on the use of animals for scientific purposes in the Member States of the European Union and Norway in 2022 2024). In most biomedical experiments, the animals are bred specifically for the experiment and are killed at the end of it and may often suffer significantly during experimentation. Therefore, ethical considerations are essential (Kolar 2006), such as the highly influential replace, reduce, refine (3R) principle (Hubrecht and Carter 2019; Russell et al. 1959). Most countries have established ethics committees specializing in animal experiments, and reporting the ethical approval for an experiment is standard in scientific publications. Group‐wise sample sizes in animal experiments are typically small in order to meet ethical requirements but also for logistical reasons, particularly restrictions on time and money. At the same time, these experiments are often purely exploratory, involving many experimental groups, so that the number of experimental groups can exceed the number of animals per group. From the first author's experience as a member of an animal ethics committee, this is more often the case than not. To add even more complexity to the experimental designs, the application process for animal studies is often lengthy and is supposed to cover future experiments over a time span of several years. Depending on the local jurisdiction, for example, in Germany, this can lead to bundling numerous sub‐experiments—sometimes involving hundreds of experimental groups—into a single proposal.

Statistical planning under such conditions is difficult and hence often informal. Formal sample size calculations are rarely conducted; instead, researchers rely on heuristics or rules of thumb. This practice leads to underpowered studies, which again affects the reliability of research results in general. Although this has been widely recognized in the methodological and applied literature, the problem continues to persist (Rowe 2023). The ARRIVE 2.0 guideline (Animal Research: Reporting of In Vivo Experiments; Du Sert et al. 2020), which has been introduced to improve the reporting quality of animal studies, places sample size justification as its first reporting item. Similarly, the PREPARE guidelines (Planning Research and Experimental Procedures on Animals: Recommendations for Excellence) emphasize the strong relationship between experimental bias and poor study design (Smith et al. 2018). Regulatory and editorial bodies have begun adopting these standards, though implementation remains uneven. Even within a single country, standards for ethical assessment and statistical requirements can vary significantly (Erdoğan and Gül 2025; Milford et al. 2025). For example, in Germany in 2020, only 8 out of 34 (∼24%) ethics committees for animal research had appointed at least one statistical expert (Piper et al. 2023). Standard requirements are still evolving; for instance, in the German city of Berlin, formal biometrical planning became mandatory for all animal research applications only in 2022.

The main approach to sample size calculation for animal experiments currently seems to be to transfer standard methodology from clinical trials to animal experiments, that is, calculating a sample size required to achieve a certain power given a desired type I error rate and effect size. Many such papers have been published and are highly cited; see, for example, Festing and Altman (2002), Fitts (2011), or Charan and Kantharia (2013). However, a classical power analysis is rarely appropriate in a field where most research is purely exploratory because even defining a primary endpoint can be challenging, not even to mention the difficulties in setting an appropriate effect size and adjusting for multiple testing.

In light of these challenges, it has been advocated to explicitly distinguish between confirmatory and exploratory research (Kimmelman et al. 2014) and there has been growing interest in reframing the goal of such studies from hypothesis testing to effect estimation, which seems more suited for exploratory research. To avoid the issues of formal power analysis, approaches have been proposed using simple rules of thumb, such as the “resource equation,” which states that the residual degrees of freedom in an ANOVA should be between 10 and 20 (Arifin and Zahiruddin 2017; Festing 2006) (but 23 would be acceptable, too, since these limits “can be liberally interpreted”; Festing 2006). Another approach is the KISS principle (Festing 2018), according to which the researcher applies “common sense” to come up with a sample size and then calculates the detectable effect size at a certain power and type I error level, and the researcher may adjust the sample size if the detectable effect size is considered to be too small.

From a methodological perspective, the choice between a strict power analysis and informal rules of thumb for the planning of complex experimental designs is clearly not satisfactory. There is an increasing number of methodological literature recommending that the analysis and the planning of animal experiments should be focused on effect size estimation instead of statistical significance (Danziger et al. 2022; Du Sert et al. 2020; Festing 2018; Kelter 2023; Piper et al. 2023; Rowe 2023). Piper et al. (2023) advocate using the width of a two‐sided confidence interval as a primary criterion for sample size justification. Similarly, the ARRIVE 2.0 guideline recommends presenting effect sizes with confidence intervals, especially when “statistical power is low and/or hypothesis testing is inappropriate” (Du Sert et al. 2020).

We agree with the aforementioned authors regarding the importance of estimation for the methodology of experimental research. However, in this paper, we argue that focusing solely on effect size estimation instead of hypothesis testing does not eliminate the statistical issues associated with multiplicity, which is a core characteristic of exploratory animal experiments. Confidence intervals and point estimates are affected by multiplicity, too, and can be misleading when multiple comparisons or endpoints are present. Thus, if effect size estimation is the stated aim, it must be accompanied by rigorous design and planning to ensure that effect estimates are both interpretable and credible. Currently, methods to estimate the estimation error of complex exploratory experimental designs are missing. In this work, we demonstrate the problem of effect size estimation in standard experimental designs and propose a simulation‐based approach to quantify the estimation error for almost arbitrarily complex experimental designs. We develop an intuitive classification of the amount of estimation error in an experimental design based on the comparison to a reference in order to facilitate communication with experimental researchers in the statistical consultation. We further propose a Bayesian error‐reducing estimator for these settings.

The remainder of this paper is structured as follows. In Section 2, we show how standard experimental designs introduce error to the effect estimates per design, even if standard approaches to multiple testing are considered. In Section 3, we develop and present the aforementioned classification of estimation error for the evaluation of experimental designs. In Section 4, we propose a shrinkage estimator based on Bayesian robust mixture priors. In Section 5, we present a simulation‐based framework to quantify the estimation error for almost arbitrarily complex experimental designs. In Section 6, we present results of a simulation study comparing various approaches to experimental designs and assessing the performance of our novel shrinkage estimators. In Section 7, we go through an example application, and in Section 8, we conclude with a summary and discussion. The methods developed in this paper have been implemented in a software which is publicly available.

## 2 Estimation Error by Design

### 2.1 Common “Standard Design” in Exploratory Animal Experiments

**FIGURE 1:**
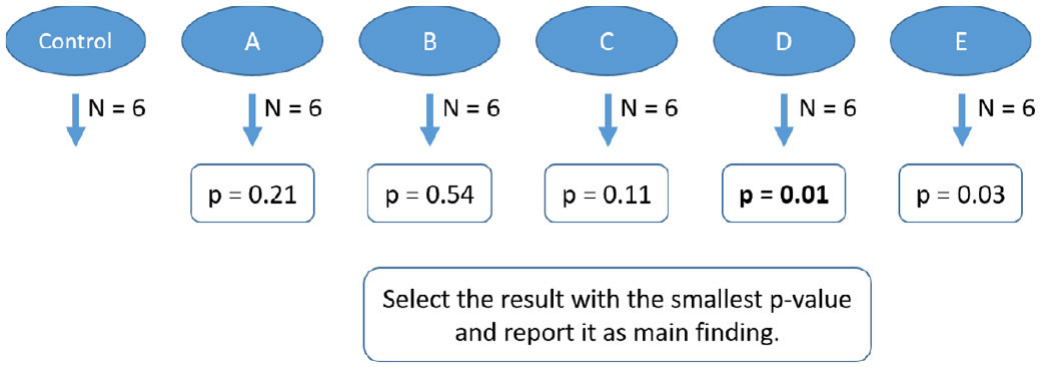
Illustration of the standard design. The experiment contains multiple (possibly many more than shown here) groups and comparisons. Statistical hypothesis testing is used to identify the largest and “most interesting” effect without any multiplicity adjustment to the effect estimate.

In experimental biology, studies are typically exploratory in nature. Hypotheses, if stated at all, are often vague, for example, stating that there will be treatment effects in one or more groups compared to a control, or entirely absent, as in the objective of detecting signals of activity in any endpoint and group. The primary goal of such exploratory experiments is to generate hypotheses based on observed data. It is common practice to selectively report only those results that align with the emerging hypotheses. Naturally, the effect sizes observed in such selectively reported data are subject to selection bias. In this work, we focus on quantifying the maximum estimation error present in an experiment and discuss statistical methods to improve its estimation. We use the term “standard design” to describe experiments that involve multiple groups and crude, unadjusted effect estimates, where the most interesting effect is selected solely based on statistical significance, without any correction for multiplicity or consideration of sequential testing procedures. (For an illustration, see Figure 1.)

Mathematically expressed, in an experiment with k groups and a continuous outcome, the null hypothesis being tested is

$$H_{0}:\mu_{1}=\mu_{2}=\cdots \;=\mu_{k}.$$

In some experiments, only a predefined set of J pairwise comparisons is of interest. In that case, it may be useful to specify the null hypothesis in terms of each pairwise difference (in the following called “effect”):

$$H_{0}:\delta_{j}=\mu_{j_{1}}-\mu_{j_{2}}=0,$$

where j∈{1,…,J} and $j_{1},j_{2}$ indicate the first and second groups of the jth pairwise comparison with corresponding two‐sided *p*‐value $p_{j}$ from Welch's *t*‐test or from a general linear hypothesis test under multiplicity adjustment. In any case, we are interested in identifying and estimating the effect associated with the smallest *p*‐value, that is, $\delta_{j^{*}}$, where $j^{*}={arg min}_{j}\left|p_{j}\right|$. As the effect estimator $\hat{\delta }$, we will apply the simple mean difference between two groups and a Bayesian estimator to be defined in Section 4. To quantify the error of an effect estimator, we will use the mean squared error (MSE):

$$\text{MSE}\left(\hat{\delta }\right)=E\left[{\left(\hat{\delta }-\delta \right)}^{2}\right].$$

Most importantly, experimental designs frequently involve multiple experimental groups, often along with their respective controls. For instance, an experiment may include genotype (e.g., wildtype and knockout), sex, age category (e.g., newborn, young, aged), and treatment (e.g., placebo vs. active treatment) as factors. If three different endpoints are measured—each requiring separate animals—the resulting design comprises 2×2×3×2×3=72 distinct groups. Such complexity is not uncommon; in fact, experiments may even include additional knockouts (especially when establishing new animal models) or examine multiple treatment doses and durations.

To give two recent examples, from *Nature Immunology*, Kwon et al. (2025) and Cembellin et al. (2025) report 7 and 14 different knockout mice, respectively, in combination with various treatments and doses. A clear description of the experimental design is missing in both papers, and so the precise number of experimental groups is unclear. Kwon et al. explicitly state that “no statistical methods were used to predetermine the sample size,” while Cembellin‐Prieto et al. do not make any statement about the determination of sample size at all.

**FIGURE 2:**
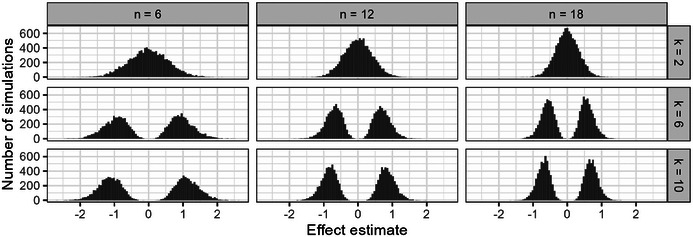
The distribution of the strongest effect estimates under the null hypothesis, that is, the true effect of 0, with the standard design approach considering different sample sizes and numbers of groups.

**FIGURE 3:**
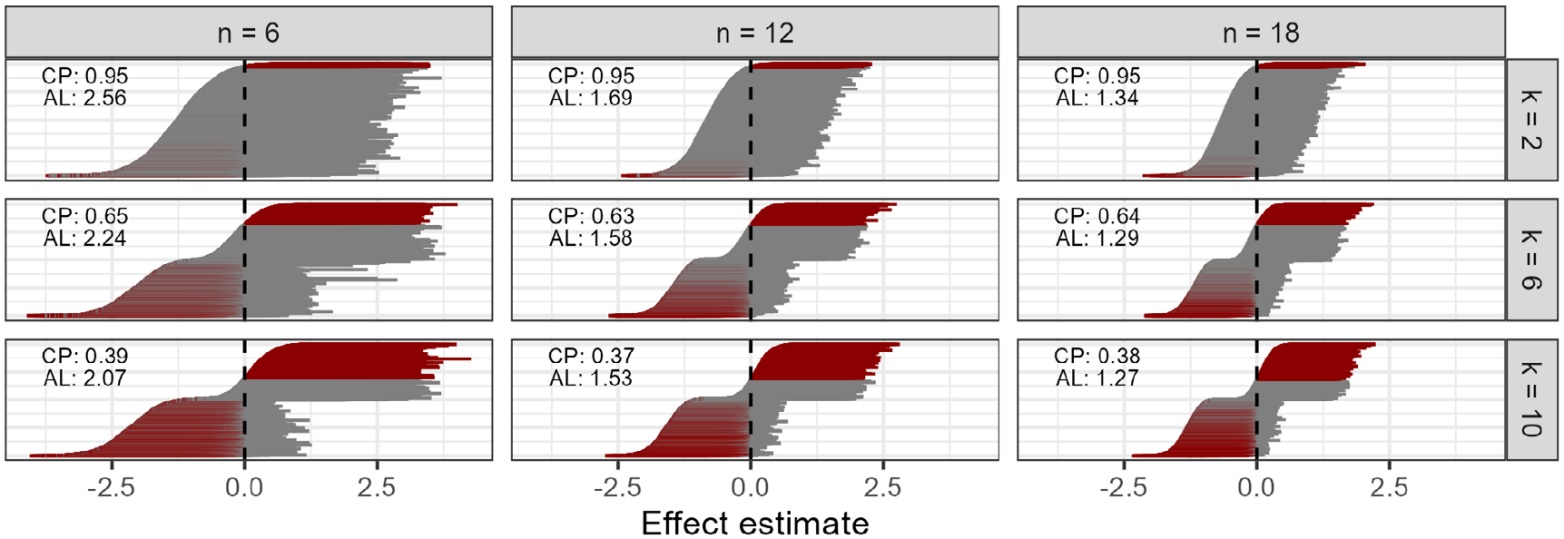
The distribution of 95% confidence intervals for the strongest effect estimates under the null hypothesis, that is, true effect of 0, with the standard design approach considering different sample sizes and numbers of groups. The confidence intervals are sorted according to their lower limit. Confidence intervals not containing the true effect are marked red. The dashed lines indicate the true value. The estimated coverage probability is denoted by CP, and the average length of the confidence intervals by AL.

This complexity has significant statistical implications. It inflates the type I error rate and introduces error into the estimated effects of the “successful” groups. While not all possible pairwise comparisons are typically analyzed, researchers often define a subset of comparisons of interest—frequently post hoc, after data collection is complete. A demonstration of the selection bias induced in the treatment effect by design is given in Figure 2, which is based on 10,000 simulation runs (for details of the simulation setup, see Section 6). With an increasing number of groups, the selected effect estimates tend to be more extreme, and the probability density at the true effect becomes effectively zero. A larger group‐wise sample size does not improve the situation: Although the size of the error is reduced, the alleged precision of the erroneous estimate is increased. The 95% confidence interval is affected by increasing experimental complexity, too, and cannot serve as a remedy for exploratory research, as recommended sometimes in the literature, for example, in the ARRIVE guideline (Du Sert et al. 2020). Not only does it lose its key property of 95% coverage probability, but it also does not accurately quantify the precision of the estimator anymore, since it is basically never centered around the true effect if the number of pairwise comparisons becomes large (Figure 3). As an indicator of the precision of the estimate, one should, in general, not rely solely on confidence intervals (Morey et al. 2016) but even less so when obtained by such an experimental design.

### 2.2 Two‐Stage Designs

In addition to the standard design, we consider two‐stage designs, in which an initial stage is used to screen for the most promising effects and a second stage is conducted to confirm them. This practice is widespread in experimental research, although it is often applied informally and without standardized protocols (Frommlet and Heinze 2021). Data from the two stages may or may not be pooled, sometimes using statistical methods that explicitly account for this pooling, such as Fisher's combination method (Frommlet and Heinze 2021) or Bayesian prior updating (Richter 2024). Importantly, such a two‐stage design is distinct from a replication study, which involves an independent attempt to replicate findings from a separate experiment. Replication studies, which have gained increasing methodological attention in recent years (Held et al. 2022; Piper et al. 2019), are outside the scope of this work, which focuses exclusively on the analysis of single (possibly multistage) experiments.

**FIGURE 4:**
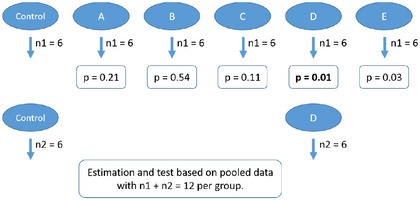
The repeat‐and‐pool design: a statistically flawed two‐stage approach, because data with different selection processes are being pooled without any proper statistical consideration. However, in practice, this approach is rather common (Frommlet and Heinze 2021). The *p*‐values are arbitrarily chosen in order to represent the logic of selecting the effect with the smallest *p*‐value and reporting the corresponding effect size as the main finding.

**FIGURE 5:**
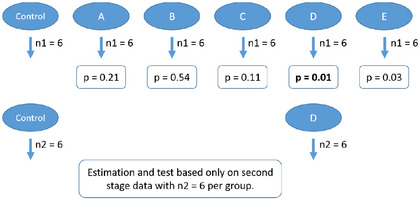
The repeat‐and‐replace design: a two‐stage approach with an obvious solution to the estimation problem, because the selection process is completely eliminated. However, all the data from the first stage is ignored for effect size estimation, which may be considered inefficient or even unethical. Also, it increases the risk of a type II error compared to the standard design and the repeat‐and‐pool design. The *p*‐values are arbitrarily chosen in order to represent the logic of selecting the effect with the smallest *p*‐value and reporting the corresponding effect size as the main finding.

We assume in this work that the first stage is used to identify the pairwise difference with the largest effect and that in the second stage only these two groups are repeatedly experimented with to confirm the estimation. We further consider two types of two‐stage designs: those that pool data from the first and second stages to arrive at an effect estimate (“repeat‐and‐pool” designs, Figure 4; whether the pooling is done statistically explicitly or not) and those that use only the second stage data for estimation (“repeat‐and‐replace” designs, Figure 5). Although there are several statistical approaches, which can potentially be used to adjust the estimates from such a two‐, and possibly multistage experimental design, none have been proposed yet for the specific requirements of complex animal experiments. Therefore, we will introduce the application of Bayesian robust mixture priors in Section 4.

### 2.3 Sample Size in Complex Experimental Designs

We understand the term “sample size” as the total number of animals to be used within an experiment, which is the product of the group‐wise sample size and the number of experimental groups. In order to allow for a fair comparison between single‐stage and two‐stage designs, we perform all calculations under the assumption that the total sample size of two‐stage designs is the same (or lower due to rounding) as the total sample size of single‐stage designs. To be precise, if n denotes the group‐wise sample size and k the number of experimental groups in the single‐stage design, respectively, then for two‐stage designs, the group‐wise sample size in the first stage is defined as $n_{1}=\left\lfloor \frac{\left(n\cdot k\right)}{\left(k+2\right)}\right\rfloor$ and the group‐wise sample size in the second stage is the remaining sample size $n_{2}=\left\lfloor \frac{n\cdot k-n_{1}\cdot k}{2}\right\rfloor$. Note that without rounding, the sample size of the second stage would monotonically increase with the increasing number of groups. However, since the sample size is rounded down, this is not the case here. Consider, for example, a design with k=9 and n=6, resulting in a total sample size of 54. In the corresponding two‐stage design, $n_{1}=4$ and $n_{2}=9$. In comparison, a design with k=10 and n=6 results in a total sample size of 60, and for the corresponding two‐stage design, $n_{1}=5$ and $n_{2}=5$.

## 3 Traffic Light System

In order to categorize a specific experimental design in terms of the amount of estimation error it produces, we propose a traffic light system. Similar approaches have, for example, been proposed for clinical feasibility studies (Avery et al. 2017; Lewis et al. 2021), but in contrast to this, the essential idea of our approach is to objectively predefine the thresholds for each category (i.e., each traffic light color) instead of leaving it up to the researchers to define these for each study individually.

We focus on the estimation error under the scenario of a true null hypothesis. There is a vast amount of literature stating that animal experiments suffer from very low power and severe publication bias (Bonapersona et al. 2021; Danziger et al. 2022; Dirnagl et al. 2022; Kelter 2023; Perel et al. 2007; Riet et al. 2012). Results from animal experiments are therefore under the general suspicion of generating false‐positive findings and overly optimistic effect sizes—to overcome this, experimental designs should first and foremost aim to provide reliable evidence under the null hypothesis.

In classical power analysis for clinical studies, the standard is to use 80% power and 5% type I error rate. Both thresholds do, in fact, not have any special meaning or mathematical justification but are mere conventions. Nonetheless, conventions are important in order to provide guidance for research projects.

Similar conventions are required when the experiments are supposed to be planned based on effect estimation, as in our proposal. As a high‐level summary, we propose to choose a reference design with no relevant risk of estimation error to classify designs with comparable or lower estimation error as GREEN (little or no error; no modifications required). If an experiment shows a larger estimation error, it is classified as YELLOW (moderate error; it is recommended to discuss possibilities to reduce the error). And if it surpasses a problematic threshold, it is classified as RED (large error; it is required to perform changes to the experimental design).

The quantification of the estimation error can be expressed as a percentage increase of the MSE compared to a reference design. The reference design should be an unbiased design, that is, the most simple design assuming the comparison of interest is known a priori. The sample size of the reference design should reflect a realistic choice in the corresponding field and should ideally be sufficient to satisfy requirements for the type I error rate and power for the two‐group comparison. However, in contrast to classical power analysis, the specification of the effect size is less crucial in our approach because it focuses on the effect estimation under the null hypothesis, that is, where there is no effect.

From this comparison, thresholds of the MSE can be defined for the three categories: GREEN, YELLOW, and RED. For the upper limit for GREEN, we suggest using the reference design. Note that with additional sequential or adaptive elements, the MSE can be further reduced, so that the reference design does not represent the experimental design with the lowest MSE possible. As to define the upper limit for YELLOW, we suggest a minimal experimental design aimed to answer a few specific, predefined research questions. This design is supposed to represent a situation where a typical statistical reviewer would not demand a multiplicity adjustment, as, for example, in a non‐confirmatory 2×2 design investigating two factors or two experimental groups compared to a control group (without interaction and without any consideration of shrinkage estimators or sequential elements). All experiments that produce larger MSE than this are considered to be in the RED zone and should therefore be subject to changes with regard to experimental design and/or analysis methods.

We admit that the threshold to distinguish the YELLOW from the RED zone is fairly subjective, and an appropriate choice may vary between fields. Recommendations on when to adjust for multiplicity vary a lot, and the choice whether to adjust or not should also depend on how results are going to be reported and interpreted (Boulesteix and Hoffmann 2024). We would like to emphasize that this subjectivity is similar to the conventional choice of 80% power and 5% type I error rate. For an example application of this traffic light system, we refer the reader to Section 7.

## 4 Bayesian Shrinkage Estimator

In the two‐stage experimental design discussed previously, the first‐stage data $D_{1}$ may be affected by selection bias, while the second‐stage data $D_{2}$ are unbiased. The error in the effect estimation in the first stage becomes smaller, the larger the true effect is, since larger true effects have a higher probability of being selected. Therefore, under the alternative hypothesis of a large effect, it is not desirable to ignore the first‐stage data for effect estimation. Instead, it would be favorable to check for agreement of first‐stage and second‐stage data regarding the effect estimate, and pool them if there is sufficient agreement.

The Bayesian framework is naturally suited to implement such a mechanism, as it allows the formal mathematical incorporation of prior data into a prior distribution, which can be assessed simultaneously with the likelihood of the second‐stage data, leading to the posterior distribution. The key challenge in this setting is the possibility of prior‐data conflicts, where the first‐stage results disagree with the second‐stage data. In this case, a naive full borrowing of the first‐stage data would be misleading since it can be subject to selection bias.

Various approaches have been proposed for similar situations in clinical studies, some of the most prominent being the ExNex (Neuenschwander et al. 2016), the power prior (Ibrahim et al. 2015), and robust mixture priors (Schmidli et al. 2014). In the following, we will present a robust mixture prior approach, of which similar versions have been applied in the clinical trials literature, but which is novel to the case of animal experimental designs. However, the other approaches are likely to be equally suitable, and more detailed research is needed in the future to determine the advantages and disadvantages of each approach.

The robust Bayesian mixture prior consists of two components: an informative component, which represents the information about the effect estimate contained in the first‐stage data, and a skeptical component, which is centered at the null effect and contributes shrinkage to the null. Such a mixture results in relatively heavy tails in the prior distribution, making it “robust” in the sense that in case of a prior‐data conflict more weight is given to the data.

Specifically, we use a linear model with intercept α to estimate the effect δ of the group indicator $x_{i}\in \left\{0,1\right\}$ on the outcome $y_{i}$ of each individual i:

$$y_{i}∼N\left(\alpha +\delta x_{i},\sigma \right).$$

The robust mixture prior for δ can be written as

$$\delta ∼\omega \times t\left(\nu ,\delta_{1},\sigma_{1}\right)+\left(1-\omega \right)\times t\left(3,\delta_{0},\sigma_{0}\right),$$

where $\delta_{1}$ and $\sigma_{1}$ are the observed mean and standard deviation of the first‐stage data, and $\delta_{0}$ and $\sigma_{0}$ are the mean and standard deviation of a skeptical prior centered at the null effect. For the informative component, we use the residual degrees of freedom ν from the *t*‐test with the first‐stage data, while for the skeptical component we set the degrees of freedom to 3 to obtain a heavy‐tailed distribution, to avoid strong shrinkage. s

The standard deviations $\sigma_{1}$ and $\sigma_{0}$ are, in frequentist terms, the “standard error” of the effect estimate, and thus we set them equal to the observed standard error of the effect estimate of the *t*‐test with the first‐stage data. In principle, $\sigma_{0}$ could be set to any reasonable value to represent a certain amount of information supporting the null hypothesis, and it should be noted that $\sigma_{0}$ must always be interpreted relative to the standard deviation of the informative component $\sigma_{1}$.

The mixing weight ω∈[0,1] determines the influence of the first‐stage data: ω=1 represents full consideration without any downweighting, while ω=0 represents complete ignorance of the first‐stage data. As long as there are no specific reasons for a specific value, we suggest using ω=0.5 as the default and testing the desired performance in simulations. If the sample sizes from both stages differ considerably, this default choice would need to be adapted accordingly.

The posterior distribution of the effect size δ is defined as proportional to the product of the prior distribution and the likelihood:

$$P\left(\delta |D_{2}\right)\propto P\left(\delta \right)\times L\left(D_{2}\right).$$

As estimator for the effect size, we use the posterior mean $\hat{\delta }=E\left(\delta |D_{2}\right)$. Similarly to the 95% confidence interval, a 95% credible interval can be obtained by taking the 2.5% and 97.5% quantiles of the posterior distribution.

An analytical solution to derive the posterior distribution under a mixture prior distribution is not available, hence we use Markov chain Monte Carlo methods, implemented in software such as Stan (2021), to obtain the posterior distribution. In Stan, the model is specified using the mixture prior above, and Stan samples from the joint posterior distribution of all model parameters using Hamiltonian Monte Carlo. The resulting posterior draws allow for uncertainty quantification, credible intervals, and decision‐making under uncertainty, all while accounting for potential prior‐data conflict in a principled and data‐driven way.

## 5 SimBA: A Simulation‐Based Framework for Estimation Error Quantification in Animal Experiments

Complex experimental designs might involve multiple groups, selected comparisons, sequential decision rules, and other features, so that calculation of the operating characteristics (the statistical properties of a design, e.g., type I error rate, power, MSE of the estimate) by closed formulae is not possible, and instead it is required to employ simulations (Meyer et al. 2025). If a simulation is appropriately implemented, almost arbitrarily complex designs can be specified and their operating characteristics can be obtained. The calculations required for our approach to the design of an experiment are also realized by means of simulation. However, an important constraint of simulations is that they require a fair amount of statistical knowledge and programming skills, as well as time for the implementation, which cannot be expected from the average experimental researcher. Therefore, we have developed a simulation software in the R programming language (R Core Team R 2024) and built an R Shiny app (Chang et al. 2025) for an accessible user interface. The following parameters are required as input from the user:

i. Number of groups,
ii. Sample size per group,
iii. Comparisons of interest,
iv. Effect size for each comparison,
v. Data‐generating distribution (either normal, lognormal, or Cauchy) with location and scale parameters,
vi. Multiple testing adjustment (for details, see the next subsection), and
vii. The weighting parameter ω∈[0,1] for the first‐stage data information in the Bayesian robust mixture prior.

As output, the simulation software estimates and generates:

i. Effect size distribution,
ii. Mean squared error,
iii. Confidence interval distribution,
iv. *p*‐value distribution,
v. Rejection rate (type I error or power), and
vi. Classification according to the traffic light system (only under the null hypothesis).

### 5.1 Multiple Testing Adjustments

Depending on the comparisons of interest, some of the following multiple testing adjustments are available: Bonferroni, Holm, Hochberg, Benjamini–Hochberg, Hommel, Dunnett, step‐down Dunnett, Tukey honest significant difference, step‐down Tukey, and the S2 method.

With Bonferroni, *p*‐values are simply multiplied by the number of comparisons, or set to 1, if the product exceeds 1.

For the Holm method, after ordering all m hypotheses by increasing p‐values, each $p_{\left(i\right)}$ is sequentially compared to an increasing sequence of thresholds $\frac{\alpha }{m-i+1}$. Testing starts with the smallest p‐value and stops at the first non‐rejection; all following hypotheses are considered to be non‐significant (Holm 1979).

The Hochberg method is a step‐up test based on Simes' inequality and can be considered a reversed Holm procedure, since it uses the same thresholds $\frac{\alpha }{m-i+1}$. Testing starts with the largest p‐value and stops at the first rejection. All hypotheses that have not been tested yet are also considered significant (Bretz et al. 2016).

The Hommel procedure is an improvement of the Hochberg procedure that uses logical constraints. For a system of m hypotheses, let S denote the set of all j∈{1,⋯,m} so that exactly j of the m hypotheses can simultaneously be true, while the remaining (m−j) hypotheses are false. Then, j is computed as $j=\max\{i\in S:p_{\left(m-i+k\right)}>\frac{k\cdot \alpha }{i}\}$ for k=1,⋯,i. If this maximum does not exist, all $H_{i}$ are rejected. Otherwise, all $H_{i}$ with $p_{i}\leq \frac{\alpha }{j}$ are rejected (Bretz et al. 2016; Hommel 1988).

The Benjamini–Hochberg method is a step‐up procedure designed to control the false discovery rate (FDR). Let $q^{*}$ denote the desired FDR level. If k is the largest i for which $p_{\left(i\right)}\leq \frac{i}{m}q^{*}$, all $H_{\left(i\right)},i=1,\text{…},k$ are rejected (Benjamini and Hochberg 1995).

The Dunnett test (Dunnett 1955) is available as both a single‐step method, which is the standard procedure for many‐to‐one comparisons, and as a step‐wise extension. The step‐down Dunnett procedure is a closed testing procedure that applies the Dunnett test to the intersection hypotheses. It is described in detail by Bretz et al. (2016).

Similar to the Dunnett test, Tukey's honest significant difference test (Tukey 1949) is available in both a single‐step version—which is a standard for pairwise comparisons—and a step‐down variant. The step‐down Tukey test proceeds analogously to the step‐down Dunnett procedure. However, due to the restricted combination condition, the corresponding closed testing procedure can be truncated. For further details, see Bretz et al. (2016).

The single‐step Tukey and Dunnett tests rely on the mvtnorm package for computing multivariate probabilities, thereby reproducing the results of the exact Edwards–Berry adjustment (Edwards and Berry 1987; Hothorn et al. 2001).

The S2 method, developed by Shaffer, is an extension of the Holm procedure. The closed testing procedure makes use of logical interrelationships between the hypotheses, thereby effectively reducing the size of the family of hypotheses to be tested in successive steps. Let $t_{i}$ denote the family size in step i. It is determined based on the specific hypotheses that have been rejected at steps 1,⋯,i−1 (Donoghue 2004). If $p_{\left(1\right)}\leq \frac{\alpha }{m}$, $H_{\left(1\right)}$ is rejected and the cardinality of the largest family of hypotheses that can simultaneously be true ($t_{i}$) is used as the Bonferroni multiplier in the next step (Westfall 1997).

It is important to note that none of these methods adjust the *estimates* for multiplicity effects but only the *p*‐values.

**FIGURE 6:**
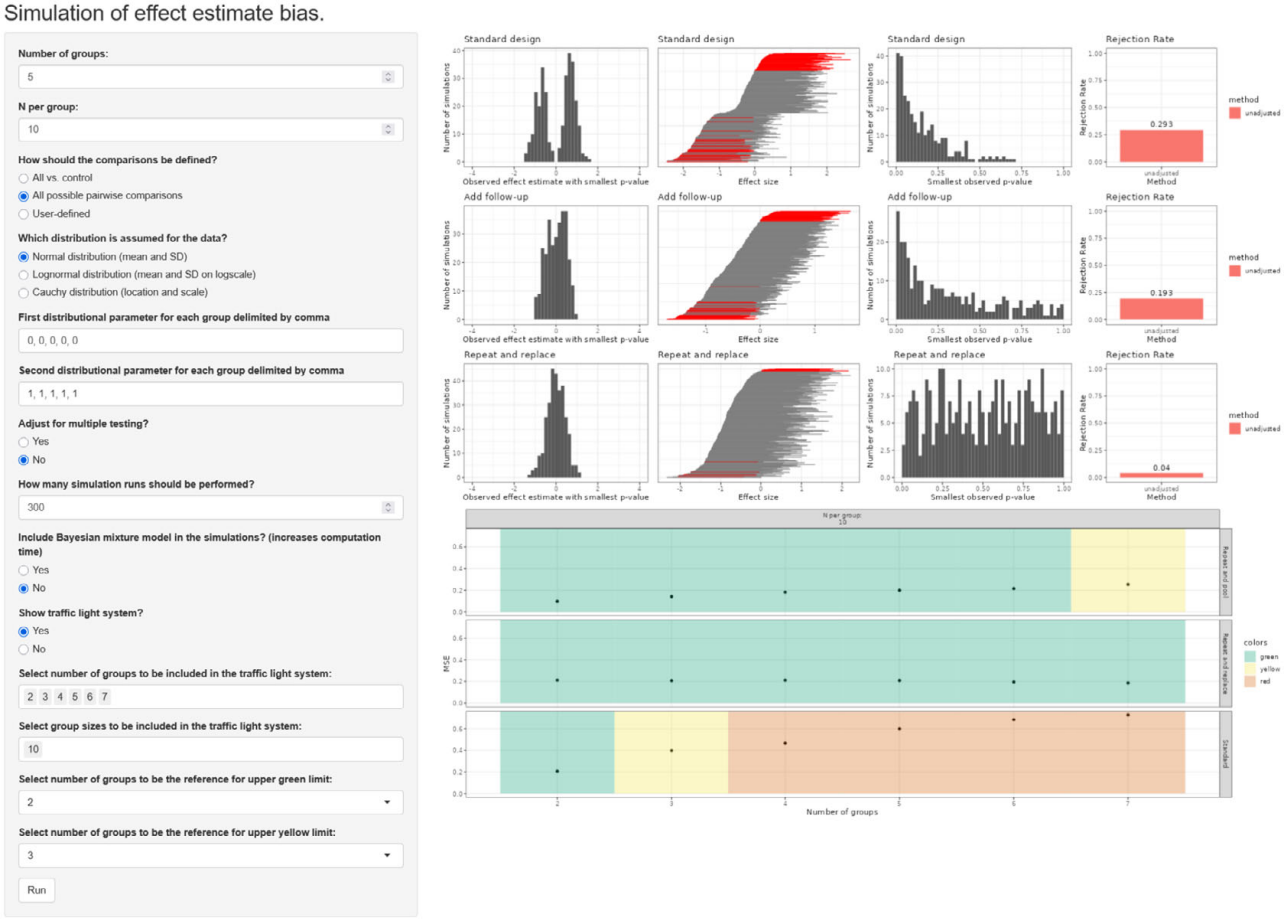
Screenshot of the SimBA shiny app.

An online demo version of the shiny app is accessible at https://dariozchl.shinyapps.io/SimBA/. A screenshot of the shiny app is provided in Figure 6. Since the simulations are quite computationally intensive, it is recommended to use the online version for small test runs only. However, that might be changed in the future, and any such changes will be documented under the same link. Currently, for any serious calculations, the user is encouraged to either download the code for the shiny app and run it on their own machine or to download the parallelized R code we used for the simulation study in this paper, modify it according to their specific experimental setup, and run it on their own multi‐core CPU. The code is available at https://github.com/dariozchl/SimBA–‐Biometrical‐Journal.

## 6 Simulation Study

### 6.1 Simulation Study Setup

In order to compare the operating characteristics of the presented experimental designs and the proposed Bayesian estimator, we conducted a simulation study. As experimental designs, the “standard design,” “repeat‐and‐pool,” “repeat‐and‐replace,” and a two‐stage design, which performs pooling with the Bayesian estimator, were considered. For the weight of the Bayesian robust mixture prior, we chose ω=0.5. The sensitivity of the approach to different choices for ω is further investigated in the Supporting Information. The “standard design” is a one‐stage design, while the other three designs are two‐stage designs, where the two groups with the pairwise difference associated with the smallest *p*‐value in the first stage are repeatedly experimented with in the second stage. All pairwise comparisons were of interest.

We assumed normally distributed endpoints with a standard deviation of 1. Effect sizes were specified in terms of the maximal mean difference $d_{\max}$: either all groups had mean 0 (the null hypothesis scenario), or all groups but one had mean 0, and the remaining group had a mean of 1 or 2 (scenarios with a true effect), thus the three effect size scenarios were $d_{\max}\in \left\{0,1,2\right\}$. Group‐wise sample sizes n were 6, 12, and 18 for the single‐stage design, and the number of groups k varied between 2 and 10. For the two‐stage designs, stage‐wise sample sizes were defined based on the total sample size n·k as described in Section 2.

In summary, there were three effect size scenarios, three group‐wise sample sizes, nine groups, and four experimental designs. Thus, in total, there were 3×3×9×4=324 simulation scenarios. Per scenario, 10,000 single‐stage and 10,000 two‐stage data sets were generated. Based on the simulated data, the effect estimates, confidence intervals, or credible intervals, respectively, and the squared error were calculated. The MSE was estimated by the mean of all simulated squared errors per scenario.

### 6.2 Simulation Results

**FIGURE 7:**
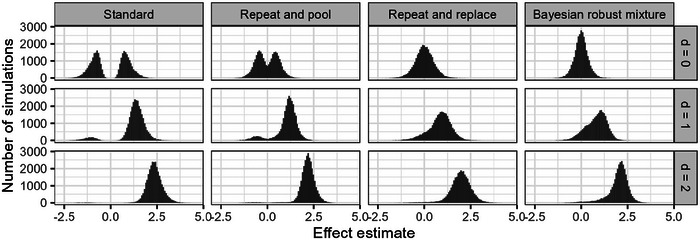
Results from the simulation study: the distribution of estimates for the strongest effect estimates under the three different scenarios for the true effect of d=0, d=1, and d=2, respectively. The four different design approaches are displayed with a number of groups k=10 and a sample size per group n=12.

The larger the number of groups in the experimental design, the larger the error in the unadjusted effect estimate from a one‐stage design. This is the logical consequence of the selection procedure and is already demonstrated in Section 2 by visualizing the effect estimate distribution under the null hypothesis (Figure 2). To illustrate the impact of the experimental design and the true effect size on the effect estimate distribution, we chose a scenario with k=10 groups and n=12 animals per group (Figure 7). In this complex experimental setup, the error in effect size estimation is quite large, and under the null hypothesis, the standard design produces a distribution with two modes and very small probability density around the true null effect. The repeat‐and‐pool design improves the estimation error by reducing the distance between the two modes. The repeat‐and‐replace design produces an unimodal symmetrical distribution around the true null effect, but with a relatively large variance because the sample from the second stage alone is rather small. In contrast, the estimates based on the Bayesian robust mixture are also symmetrical and unimodal around the true null, but with much smaller variance. In the scenario with a moderate effect size of d=1, estimation of the standard design becomes much better because most of the time the groups with the strongest effect are correctly identified. The difference to the repeat‐and‐pool design gets small. The effect estimate distributions of the repeat‐and‐replace design and the Bayesian approach are both not perfectly symmetrical anymore, because of the (rather small) proportion of wrongly selected pairwise comparisons. Additionally, the shrinkage component in the Bayesian mixture prior results in the variance being larger than in the repeat‐and‐pool approach. In the scenario with a moderate effect size of d=2, the estimation problem is less affected by selection bias because, almost always, the correct groups are identified. In this situation, the standard design can be considered as unproblematic; however, the two‐stage designs produce similar effect estimate distributions, with estimates from the repeat‐and‐pool design having even smaller variance, estimates from the Bayesian approach being very similar but containing a small shrinkage effect, and the repeat‐and‐replace approach showing a somewhat larger variance.

**FIGURE 8:**
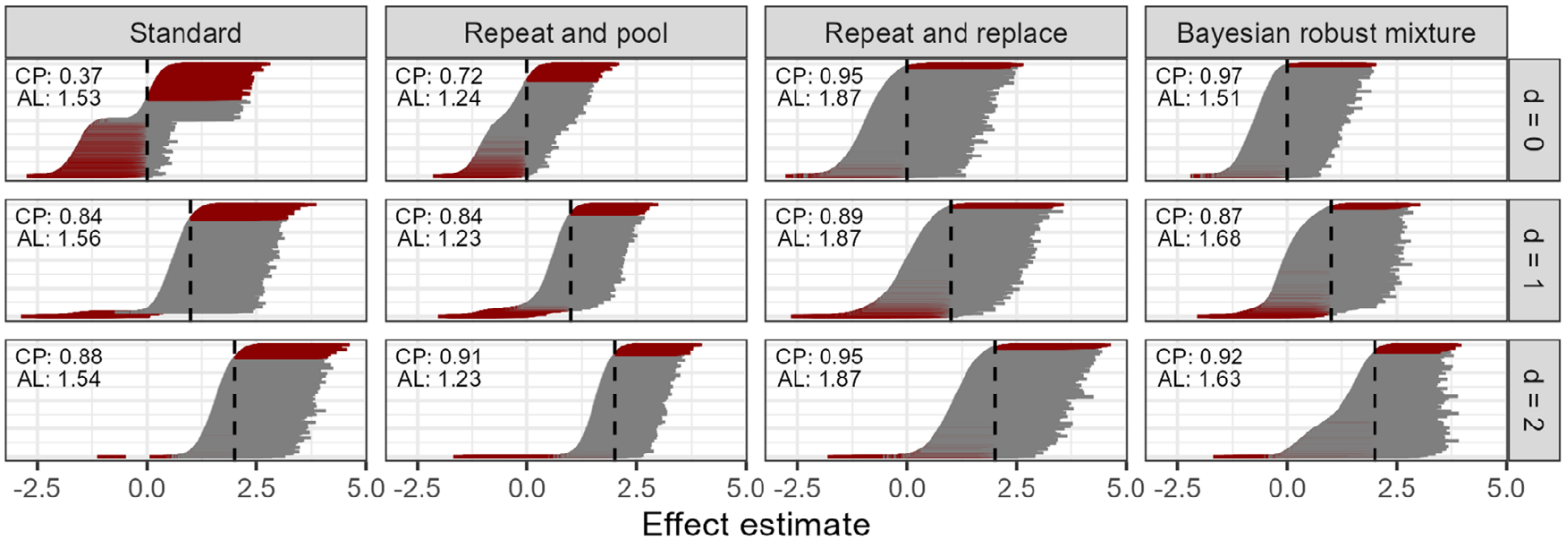
Results from the simulation study: the distribution of 95% confidence and credible intervals for the strongest effect estimates under the three different scenarios for the strongest effect, that is, true effect of d=0, d=1, and d=2, respectively. The four different design approaches are displayed with the number of groups k=10 and sample size per group n=12. The confidence intervals are sorted according to their lower limit. Confidence intervals not containing the true effect are marked red. The dashed lines indicate the true value. The estimated coverage probability is denoted by CP, and the average length of the confidence intervals by AL.

The distribution of confidence and credible intervals (Figure 8) reveals similar observations as already described in the effect estimate distributions. While the distribution under the null hypothesis is highly bimodal for the standard design, with basically no confidence interval centered around the true null effect, the distribution is symmetric and centered around the true null effect for the two‐stage approaches. The repeat‐and‐pool design has the narrowest confidence intervals, but also has an inflated proportion of confidence intervals not containing the true null effect. For larger effect sizes, the standard design becomes less problematic, but the repeat‐and‐pool approach might still be favorable because of its narrower confidence intervals. Notably, the robustness of the mixture priors becomes particularly apparent in the scenario for a larger effect size with d=2: for larger effect estimates, the 95% credible intervals are relatively narrow because the prior information from the first stage is assigned more weight, while for effect estimates closer to 0, the informative component is downweighted compared to the data. This increases the uncertainty in the estimate and widens the 95% credible intervals.

**FIGURE 9:**
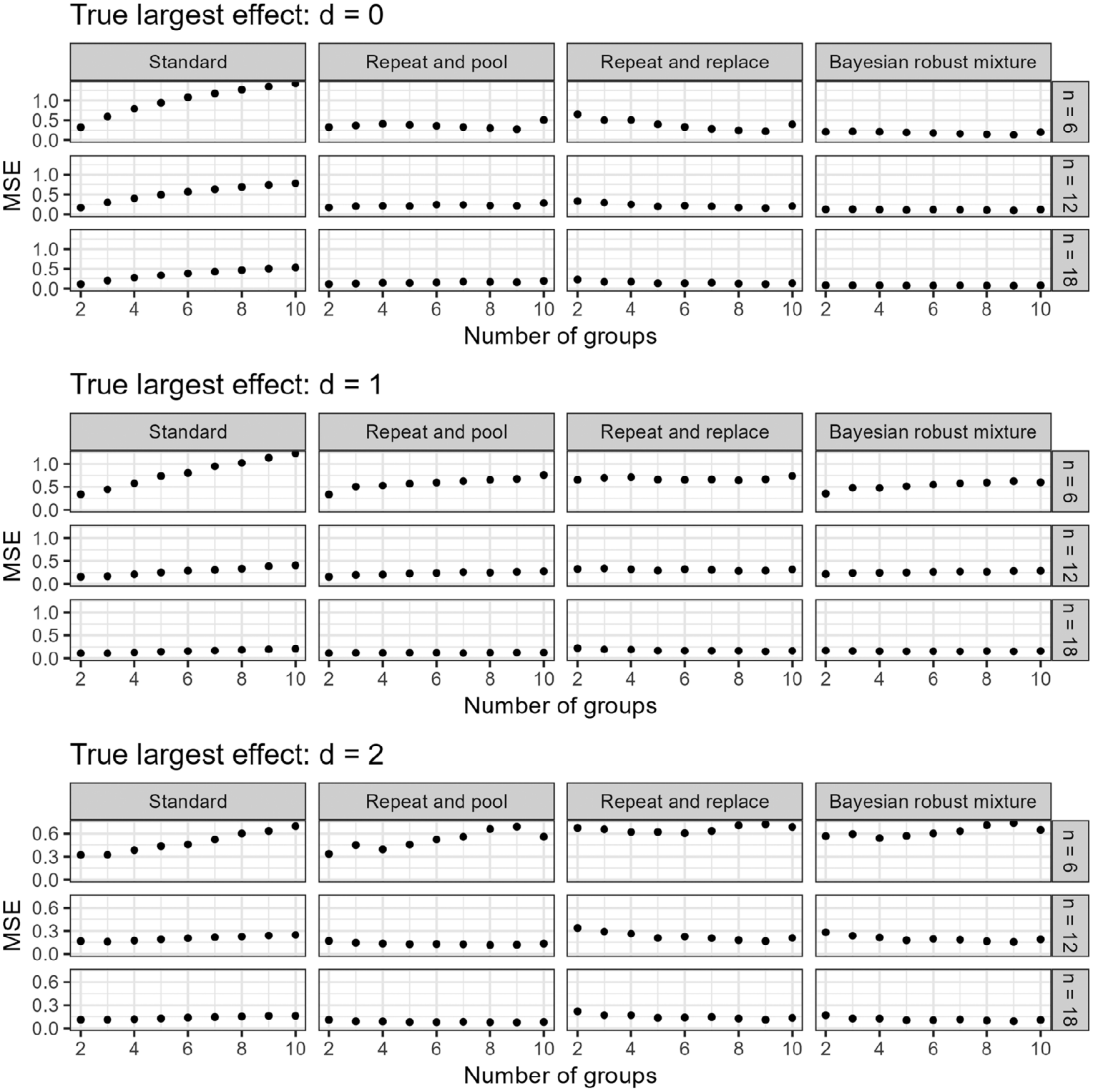
Results from the simulation study: mean squared error based on 10,000 simulation runs per scenario.

The estimates for the MSE for all simulation scenarios are shown in Figure 9. Clearly, under the null hypothesis, the standard design produces the largest error for almost all scenarios, which further increases with the increasing number of groups. Larger group‐wise sample sizes decrease the error in absolute terms; however, the relative difference between design approaches and number of groups is not affected. With increasing effect size, the error of the standard design reduces—if the effect size becomes so large that the selection bias practically vanishes (as is the case for d=2), the standard design even becomes the most efficient approach in some scenarios. However, all designs perform similarly, and the number of groups also does not have a relevant impact anymore.

**FIGURE 10:**
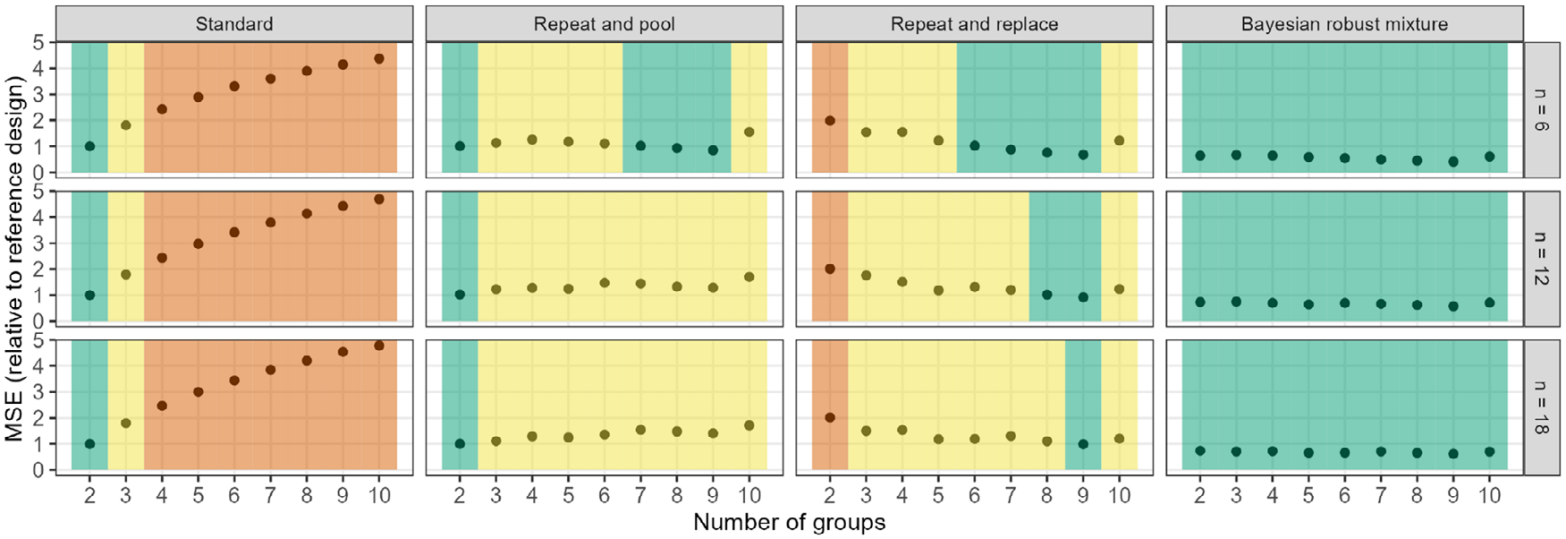
Results from the simulation study: an application of the traffic light system (colors optimized for color‐blind readers; Wong 2011).

Finally, from the MSE under the null hypothesis, we derive the categorization according to the traffic light scheme (Figure 10). For this, the MSE of each design specification was set in relation to the MSE of the reference designs, that is, the standard design with two groups. All designs with relative MSE smaller than 1 (with an additional 5% margin of error) were categorized as GREEN (“unproblematic”). All designs with relative MSE larger than the threshold for GREEN, but smaller than the standard design with three groups (again with 5% upper margin of error) were classified as YELLOW (“acceptable”). All designs with larger MSE were classified as RED (“requires modification”). According to this classification, the repeat‐and‐pool design still obtains acceptable MSE with many more groups than the standard design approach. Under the Bayesian design approach, all specifications are classified as GREEN, demonstrating the superior effect estimation of the approach in the null hypothesis scenario. The repeat‐and‐replace design is not affected by selection bias, still the MSE is in the yellow or even red zone, because it considers second‐stage data only and does not employ any pooling, leading to high variance in the estimation. Although being counterintuitive at first, the MSE of two‐stage designs is not monotonically increasing or decreasing with increasing number of groups. This is because the second stage sample size is not monotonically increasing or decreasing with increasing number of groups due to rounding (see Section 2 for a more detailed discussion).

For the reasons provided in Section 3, we focus on the null hypothesis here. Results for the traffic light system under the two alternative hypotheses are provided in the Supporting Information.

## 7 Example Application

To illustrate the application of our approach and the traffic light system in particular, we analyzed an anonymized version of an actual ethics application that was submitted for review to the ethics committee, of which the first author of this paper is a member. The experiment aims to evaluate neural mechanisms of chemotherapy‐induced side effects. The total sample size is 1067, which is split up into 13 subexperiments, the largest of which comprises 216 animals with 9 animals per group, corresponding to 24 treatment groups. Focusing on this subexperiment, there are 6×2×2 factors: six different ways to physically manipulate the neural function, the corresponding control without physical manipulation, and chemotherapy versus control. Additionally, mice from both sexes are pooled, but the effect of interaction with sex may be possible. Sample size calculation is based on a reported effect size of approximately d=1.4 for a pairwise *t*‐test. Due to the exploratory nature of the experiment, there is no adjustment for multiplicity and no predefined set of comparisons—instead, Cohen's *d* and confidence intervals of mean differences for all pairwise differences are going to be reported, resulting in 24!/(2!·(24−2)!)=276 effect estimates.

The multiplicity issue in this experiment is even larger than considered in our simulations. However, it is likely that in a careful statistical consultation, the number of pairwise comparisons could be limited. As an analysis method, a factorial design is probably better suited than simple pairwise comparisons, which further reduces the number of effect estimates required. The necessity of such a prespecified limitation of the analysis can be demonstrated to the researchers by referring to Figure 10, where even with only 10 groups the MSE of the best effect estimate in the proposed standard design is increased fourfold compared to a simple two‐group comparison, and their design is far in the red area, indicating a highly problematic effect estimation strategy.

Depending on the researcher's aims, one of two recommendations might be useful here. If the researchers want to identify which of the six physical manipulations is the most beneficial, then effect estimation is the analysis goal. In this situation, a multistage approach with appropriate pooling for confirmation of identified effects, possibly using Bayesian robust mixture priors, will be the most suitable approach. If instead the researcher's aim was to investigate the interactions of each physical manipulation with the chemotherapy and sex, then a significance testing strategy in a factorial design could be more appropriate. A power analysis for such a complex design would not be straightforward due to the required specification of interaction effects, but could be implemented in our SimBA framework (requiring some additional coding for the factorial design).

Ideally, the consideration of multiplicity in the effect estimation during the planning phase would be documented in a preregistration of the (sub‐)experiment (Van Der Naald et al. 2022).

## 8 Discussion

Our approach addresses a well‐known dilemma in statistical consulting for research projects. Involving statistical expertise and applying classical power analysis methods in the planning of experiments usually leads to the recommendation of larger group‐wise sample sizes, but the reliability of the experimental data would actually increase if experiments would tend to be smaller, that is, less complex and more focused to be well equipped to quantify specific endpoints. So far, there are no approaches available to statistically assess the magnitude of error in the effect estimates introduced by complex designs. With our approach and the accompanying simulation software, the effect estimation error can be quantified and effectively communicated to the researchers. Specifically, we provide guidance on how to improve problematic experimental designs. The proposed traffic light system provides an intuitive way to objectively classify an experimental design and indicates where improvement of the design is required.

It should be noted that our approach does not replace the classical approaches to determine group‐wise sample sizes, but complements them by considering the complexity of the whole experimental design. The group‐wise sample size would still need to be determined based on other factors, such as a power analysis, the desired width of confidence intervals, or established standards in the field. However, our proposed simulation framework can also be used to perform these calculations, and, in contrast to common sample size calculation software, this can be done even for complex experimental designs.

We have also proposed a shrinkage estimator for two‐stage experiments based on Bayesian robust mixture priors. The estimator incorporates information from the first stage data into an informative component of the prior distribution, which is complemented by a skeptical component centered at the null effect. This estimator reduces the error in the effect estimators under the null hypothesis, while causing moderate shrinkage towards 0 if a true effect exists. A weighting parameter can be tuned to give either more weight to the prior information or to the skeptical assumption. In principle, this approach can be flexibly applied to any effect estimate, not only to the strongest effect selected in the first stage. It should be noted that there are several approaches, both frequentist and Bayesian, with which similar behavior could probably be achieved. Our aim was not to recommend a specific estimator as the “best” solution according to some optimality criterion, but to demonstrate the application of the traffic light scheme by using a rather simple approach to improve estimation.

Compared to clinical trials, there is still a lot of room for improving the statistical planning and analysis of animal experiments, and, currently, our approach does not consider several aspects that are standard to clinical trial methodology. For example, in many experiments, cluster effects should be taken into account properly, for example, because animals may stem from the same litter, are kept in the same cage, or are measured multiple times. There is increasing methodological research on multistage designs and platform trials, and some of their aspects, such as group‐sequential designs, shared controls, and Bayesian decision rules, may be well applicable to animal experiments (Meyer et al. 2025). There are multiple statistical methods for the combination of data from different experimental stages that were not considered here, such as Fisher's method or mixed‐effects models (Frommlet and Heinze 2021). In adaptive clinical trials methodology, multiple bias corrections for point estimates and confidence intervals in two‐stage trials have been developed (Grayling and Wason 2023; Meis et al. 2024) but these would need to be specifically tailored for exploratory experimental designs since they do not adjust simultaneously for the bias due to sequential testing and for the bias due to comparing many groups. We aim to expand our approach to consider these aspects in the future.

Further potential lies in the possibility of incorporating multiple, preferably only weakly correlated, endpoints and analyzing them in a multivariate model. Implicitly, it is a common approach to use multiple endpoints to obtain a “complete picture” of a pathophysiological mechanism, but its appropriate consideration by statistical modeling and planning is still open research. Another approach to reduce the number of animals required, which has not been considered in our work, is the use of historical controls, which can be methodologically incorporated by applying the Bayesian framework (Bonapersona et al. 2021).

An important limitation of this work is the focus on continuous outcomes and parametric ANOVA as a statistical model. Nonparametric analyses are common in experimental research, and it might be interesting to investigate how these could be applied in the context of effect estimation. Although less common, the consideration of binary, count, ordinal, or time‐to‐event outcomes would be rather straightforward and could be another valuable extension of our work. Furthermore, we have focused on pairwise comparisons, but there are good arguments to use factorial designs in multiple experiments (Grüninger and Frommlet 2024), and integrating factorial models in our approach could be a worthwhile direction for future research.

## Conflicts of Interest

The authors declare no conflicts of interest.

## Open Research Badges

This article has earned an Open Data badge for making publicly available the digitally‐shareable data necessary to reproduce the reported results. The data is available in the Supporting Information section.

This article has earned an open data badge “**Reproducible Research**” for making publicly available the code necessary to reproduce the reported results. “The results reported in this article could fully be reproduced.”

## Supporting information


**Supporting File 1:** bimj70116‐sup‐0001‐SuppMat.pdf.

**Supporting File 2:**bimj70116‐sup‐0002‐Datacode.zip.

## Data Availability

The data that support the findings of this study are openly available in GitHub at https://github.com/dariozchl/SimBA–‐Biometrical‐Journal.
