# Supplementary material for: A Novel Approach to the Design and Sample Size Planning of Animal Experiments Based on Effect Estimation
Source: Biom J. 2026 Mar 15;68(2):e70116. doi: 10.1002/bimj.70116 (PMC12989740; doi:10.1002/bimj.70116)
Supplement: Supplementary file 1 — Supporting File 1: bimj70116‐sup‐0001‐SuppMat.pdf. [file BIMJ-68-e70116-s001.pdf]

Supplementary materials for

A novel approach to the design and sample size planning of animal experiments based on effect estimation

1 Different weights for Bayesian robust mixture priors

Here, we present simulation results for various weights for the Bayesian robust mixture priors (Figure 1). Recall that we define the mixture priors as

$$\delta \sim \omega \times t(\nu, \delta_1, \sigma_1) + (1 - \omega) \times t(3, \delta_0, \sigma_0),$$

so ω refers to the weight of the first stage information. Note that the sample size of the first stage information is reflected in the prior parameters σ_1 and σ_0, so for a proper interpretation of ω, one should set $\sigma_1 = \sigma_0$. We have performed the simulation from the main paper also for mixture weights $\omega \in \{0.25, 0.5, 0.75\}$ to demonstrate the impact of the choice of the mixture weights. If $\omega = 0.25$, the data from the first stage receives less weight and the skeptical component is attributed more weight, if $\omega = 0.75$ it is the other way round.

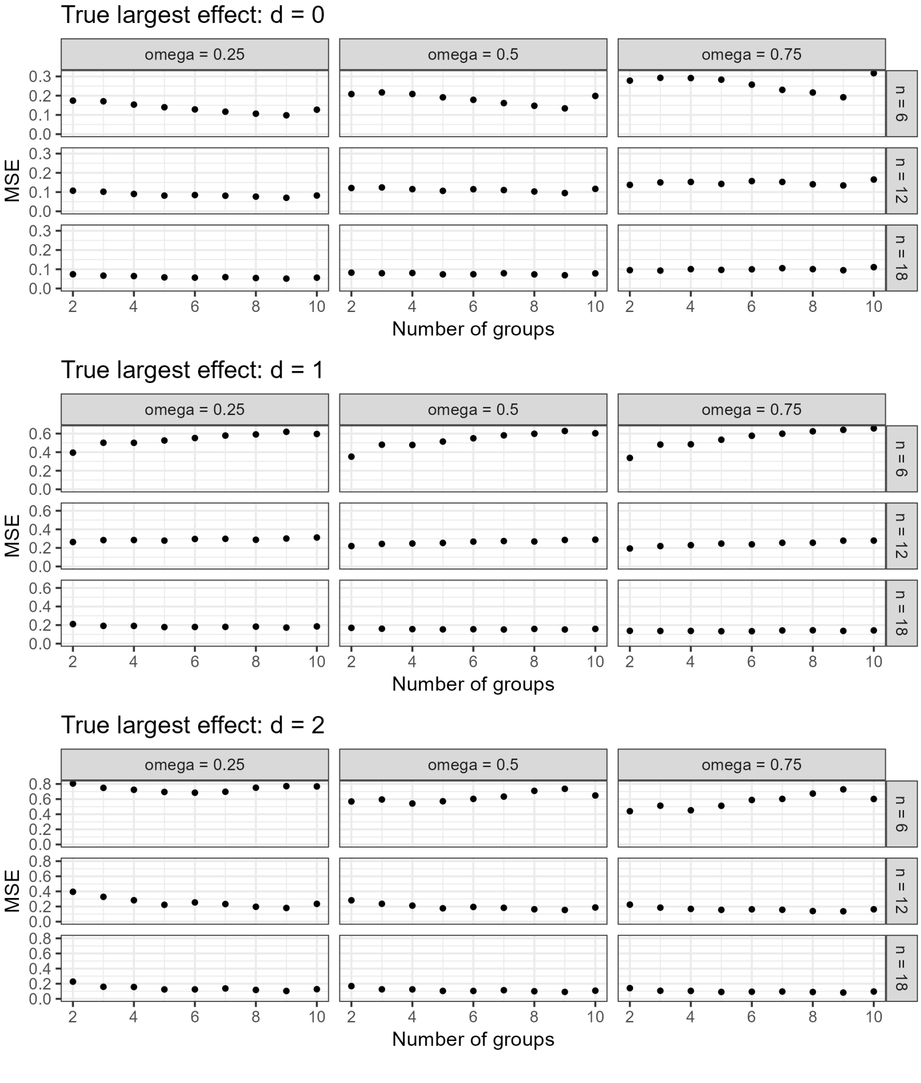

Figure 1: Estimated mean squared error of the effect estimator for different values for for various scenarios based on 10,000 simulation runs.

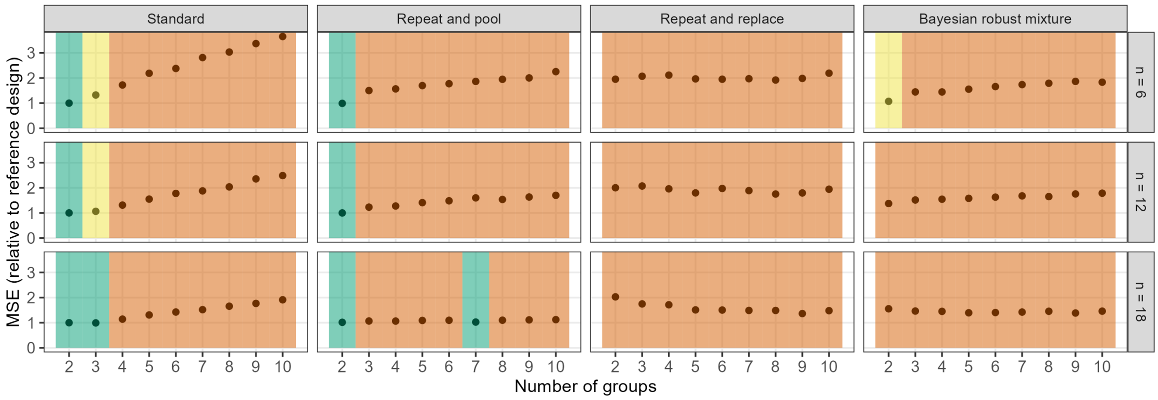

Figure 2: An application of the traffic light system in analogy to Figure 10 in the main manuscript, but under the alternative hypothesis d = 1.

In Figure 1 it can be seen that the MSE is smallest for $\omega = 0.25$ under the null hypothesis, which is due to the skeptical component, which shrinks estimation towards 0, is given more weight. Under the alternative hypothesis $d = 1$, virtually no differences can be seen between the different mixture weights. This is because in this scenario, there is still considerable selection bias, i.e., the effect size estimates from first stage are inflated, but the skeptical component contains wrong information, too. So, neither component is correct, and hence different weights only affect the direction of the error but not its magnitude. Under the alternative hypothesis $d = 2$, MSE is the smaller the larger ω is, reflecting the value of the first stage data, because in this scenario there is very little selection bias.

These effects are much more pronounced in scenarios with small group-wise sample size, because there the error is larger in general and differences become more notable due to the scaling.

2 Traffic light system under the alternative hypothesis

Of course, the idea of presenting the relative MSE for various designs can be applied both under the null and under any alternative hypothesis. Such an analysis is provided in Figures 2 and 3. In this analysis, most designs are in the RED zone. The notable exception is the repeat-and-pool design under the alternative hypothesis $d = 2$. However, it should be noted that under a strong alternative hypothesis, multiplicity does not affect estimation anyways, so it is a logical consequence that the selection bias is almost eliminated and thus pooling the data from both stages is statistically efficient.

We want to emphasize that in the process of a statistical consultation with applied experimental researchers, such information could give the impression that under the desired large effect sizes, standard experimental designs which ignore multiplicity would not pose any statistical issues. As we write in our manuscript, there is a lot of evidence that experimental literature suffers from problems caused by multiplicity, and hence reliable effect estimation under the null hypothesis should be the priority in our view.

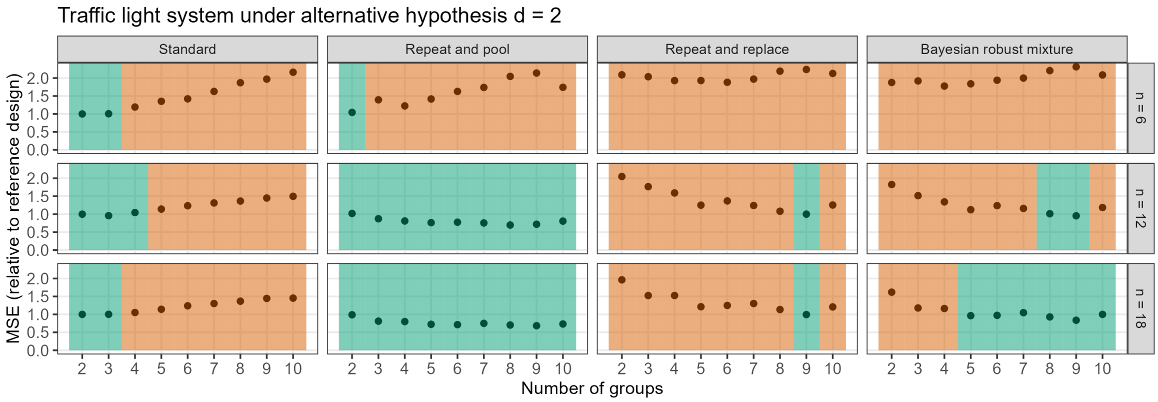

Figure 3: An application of the traffic light system in analogy to Figure 10 in the main manuscript, but under the alternative hypothesis d = 2.
